# Knowledge, belief, and practice in prevention of lymphedema in postoperative breast cancer patients and analysis of associated factors

**DOI:** 10.3389/fpubh.2025.1474419

**Published:** 2025-06-02

**Authors:** Qinqin Li, Yunli Yan, Ying Luo, Xiaoli Chen

**Affiliations:** ^1^School of Nursing, Wuhan University, Wuhan, Hubei, China; ^2^Breast Cancer Center, Hubei Cancer Hospital, Tongji Medical College, Huazhong University of Science and Technology, National Key Clinical Specialty Discipline Construction Program, Hubei Provincial Clinical Research Center for Breast Cancer, Wuhan Clinical Research Center for Breast Cancer, Wuhan, Hubei, China; ^3^Beihu Community Health Service Center, Wuhan, Hubei, China

**Keywords:** breast cancer, lymphedema, knowledge, belief, practice, influencing factors

## Abstract

**Purpose:**

To investigate the knowledge–belief–practice (KBP) regarding lymphedema prevention among postoperative breast cancer patients and identify its psychosocial determinants.

**Methods:**

Postoperative patients were selected using a convenience sampling method. A general information collection, questionnaires, a Chinese version of the Distress Disclosure Index (DDI), and the Hospital Anxiety and Depression Scale (HADS) were used. Multivariate linear regression was used.

**Results:**

The total scoring rate of knowledge, belief, and practice was 58.51%, with the lowest for knowledge and highest for belief. The level of knowledge, belief, and practice was positively correlated with self-representation and negatively with anxiety and depression. The multivariate linear regression showed that receiving health education on the knowledge, family income, anxiety, depression, and self-expression levels were the critical factors influencing lymphedema.

**Conclusion:**

The level of knowledge, belief, and practice is at the lower-to-middle level in China, with poor knowledge mastery, and the level of practice needs to be improved. Healthcare personnel should conduct health education to improve patients’ knowledge level related to lymphedema and enhance the correct health beliefs of the patients. Meanwhile, they should also pay attention to their psychological health status to help them improve the level of self-expression and carry out personalized interventions according to the influencing factors.

## 1 Introduction

According to the Global Cancer Statistics 2022 released by the International Agency for Research on Cancer (IARC), breast cancer accounted for 2.297 million new cases worldwide, representing 23.8% of all female cancer diagnoses and ranking as the second most prevalent malignancy across both genders combined, while maintaining its position as the leading cancer type among women. Notably, China reported 357,000 new breast cancer cases in 2022, constituting 15.6% of female cancer cases nationally and 15.5% of global female breast cancer incidence. This positions breast cancer as the sixth most common malignancy in China’s general population and the second most prevalent among Chinese women (1). Emerging as a significant public health concern, breast cancer now stands as one of the primary common malignant tumors worldwide, posing significant threats to women’s health. The advent of precision medicine and multidisciplinary treatment paradigms has catalyzed the development of novel diagnostic modalities and therapeutic approaches, resulting in a marked improvement in 5-year relative survival rates to 91% (2). However, this epidemiological success has been accompanied by new challenges: as breast cancer prevalence continues its upward trajectory, increasing attention is being directed toward understanding and improving quality of life outcomes for this growing patient population.

Current therapeutic paradigms for breast cancer are mainly based on multimodal approaches centered around surgical intervention, which often lead to a range of treatment-related complications. Of particular clinical significance, breast cancer-related lymphedema (BCRL) emerges as the most prevalent intermediate-to-late postoperative sequela, constituting a principal determinant of long-term quality-of-life impairment in survivors. Longitudinal epidemiological surveillance reveals a progressive accumulation pattern, with cumulative BCRL incidence rates escalating from 13.5% at the 2-year postoperative mark to 30.2% by year 5, ultimately reaching 41.1% at the decade milestone, as evidenced by a 10-year prospective cohort study (3).

BCRL may lead to both physiological and psychological complications, including impaired limb mobility, body image disturbances, anxiety disorders, and clinical depression (4). This condition not only increases healthcare costs and financial strain for patients but also significantly undermines postoperative rehabilitation outcomes and overall quality of life. Characterized by progressive deterioration, BCRL manifests as an irreversible pathological state with no definitive cure currently available, requiring patients to adopt lifelong self-management strategies. These clinical realities highlight the essential role of preventive measures and early-stage therapeutic interventions for effective lymphedema management (5).

While qualitative investigations into lymphedema prevention among breast cancer patients have been conducted, significant gaps remain in understanding postoperative patients’ knowledge, attitude, and behavioral practice regarding preventive measures. The multivariate meta-analysis performed by Wang et al. identified personal self-management competencies as a critical determinant, although substantial interstudy heterogeneity and methodological confounding limited the conclusiveness of findings (6). This observation aligns with epidemiological research on podoconiosis in Rwanda, which established strong correlations between disease outcomes and patients’ health literacy, belief systems, and preventive behaviors (7). To address these evidence gaps, our study systematically evaluates the current landscape of lymphedema prevention through the tripartite lens of the knowledge–belief–practice (KBP) framework. Through comprehensive factor analysis, we aim to establish an evidence base for developing precision nursing interventions that optimize health outcomes in postoperative breast cancer populations.

## 2 Objects and methods

### 2.1 Objects of study

This cross-sectional investigation included breast cancer survivors from five tertiary care hospitals in Hubei Province between August 2023 and November 2023. Participants were selected based on stringent inclusion criteria: (1) postoperative breast cancer patients who were diagnosed with breast cancer by histologic pathology and had undergone axillary lymph node dissection; (2) aged ≥ 18 years; (3) possessing normal intelligence level and ability to communicate verbally or in writing; (4) having signed informed consent and voluntarily participating in this study. Exclusion parameters included: (1) individuals with severe cognitive impairment or psychiatric disorders; (2) individuals with other serious somatic diseases or malignant tumors; (3) current enrollment in lymphedema intervention trials. The sample size was calculated using the multifactorial analysis heuristic: N = independent variables × (5–10) times (8), With 18 predictor variables analyzed and a 20% anticipated attrition rate, the required sample range was determined to be 108–216. To ensure statistical robustness, we ultimately enrolled 321 eligible participants, exceeding the upper threshold of our initial estimates. This study was approved by the Ethics Committee of Hubei Provincial Cancer Hospital (LLHBCH2023YN-031), and the study sample was authorized by the same institution. All study subjects were informed about the study and participated voluntarily.

### 2.2 Methodology

#### 2.2.1 Data collection and quality control

The survey was conducted in the form of a web-based questionnaire, with the help of the Questionnaire Star platform^1^ to generate the questionnaire link and a quick response (QR) code, and the survey platform was open from August to November 2023. The questionnaire was completed anonymously and individually by the patients. If the respondents had any questions about the questionnaire’s content, the investigators provided one-on-one communication and guidance on the spot. The investigators communicated and explained based on the principle of neutrality. The questionnaire is strictly set up for each Internet Protocol (IP) address and can only be completed once. All entries are mandatory to ensure the completeness of the questionnaire. Questionnaires with a total answer time of less than 2 min or unreasonable answers were excluded when the questionnaires were recovered to ensure the quality of questionnaire completion. This study adhered to the principles of voluntariness, confidentiality, and non-harmfulness. Participants were considered to have agreed to participate in the study if they completed and submitted the questionnaire.

#### 2.2.2 Survey instruments

##### 2.2.2.1 Questionnaire of patient general information

Self-designed by the researcher according to the research purpose and concerning the relevant studies, this study specifically included age, place of residence, education level, work status, per capita monthly household income, marital status, payment method, primary caregiver relationship, number of postoperative hospital admissions for breast cancer, whether or not lymphedema had occurred, whether or not they had received health education on lymphedema prevention, and ways to obtain knowledge on lymphedema prevention.

##### 2.2.2.2 Knowledge and belief scale for prevention of upper extremity lymphedema in postoperative breast cancer patients

This scale was developed by Bohui et al. (9). This scale was developed using the Knowledge, Belief, and Practice Theory as a framework, and consists of 23 entries in three dimensions, all of which are single-choice questions, including knowledge (10 items), belief (6 items), and practice (7 items), of preventing lymphedema in patients with postoperative breast cancer. Each entry was scored on a 5-point Likert scale, “do not know at all/do not agree at all,” “do not know much/do not agree,” “know some/agree more,” “Understand/Agree,” and “Know Very Much/ Agree,” or “Never,” “Occasionally,” “Sometimes,” “Often” and “Always,” to assign a score of 0, 1, 2, 3 and 4, without reverse entry. The total score is the sum of the entries, ranging from 0 to 92, and the higher the score, the better the level of prevention of lymphedema in postoperative breast cancer patients. The raw scores were converted into percentages to calculate the scoring rate, and a scoring rate of <60% was regarded as a low level, 60–79% was regarded as a medium level, and ≥80% was regarded as a high level. The content validity of each entry on the scale was 0.857–1.000, and the content validity of the total scale was 0.938. The results of the reliability analysis showed that the Cronbach’s alpha coefficient of the scale was 0.931, the Split-half Reliability Coefficient was 0.875, and the re-test coefficient was 0.916. The scale has good reliability and validity and can be used as an assessment tool for patients’ knowledge, belief, and practice of lymphedema prevention.

##### 2.2.2.3 Chinese version of distress disclosure index (DDI)

The scale was developed by Hessling et al. and revised by Chinese scholar Li (10). The scale has a total of 12 entries, using Likert 5-level scoring, “never,” “occasionally,” “sometimes,” “often,” and “always,” of which 1, 2, 3, 4, and 5 points are assigned. Entries numbered 2, 4, 5, 8, 9, and 10 are deemed reverse-scoring entries, and each holds a value ranging from 5 to 1 points. The total score was 12–60 points, and the higher the score, the higher the individual’s self-expression level. 12–29 points was a low level, 30–44 points was a medium level, and 45–60 points was a high level. The scale’s Cronbach’s alpha coefficient was 0.887.

##### 2.2.2.4 Hospital anxiety and depression scale (HADS)

The scale was developed by Zigmond and Snaith (11). The scale consists of 14 items, divided into 2 subscales for anxiety and depression, with 7 items for depression and 7 items for anxiety, and each rated on a 4-point Likert scale from 0 to 3. The sum of the scores of each entry of the two subscales was the final score. The total scores of the subscales ranged from 0 to 21, with 0–7 being asymptomatic, 8–10 being mild anxiety and depression, 11–15 being moderate anxiety and depression, and 16–21 being severe anxiety and depression. The Cronbach’s alpha coefficients of the overall HADS, the anxiety subscale, and the depression subscale were 0.879, 0.806, and 0.806, respectively.

#### 2.2.3 Statistical methods

This study used SPSS 25.0 for statistical analysis. The count data were described by frequency and percentage, and the measurement data conforming to normal distribution (Shapiro–Wilk test was used to test for normality) were characterized by mean ± standard deviation (*x* ± SD). Two independent samples’ *t*-test or ANOVA were used to compare the differences in the scores of the knowledge, belief, and practice regarding the prevention of lymphedema in patients with different postoperative breast cancer characteristics.

The factors influencing the level of knowledge, belief, and practice scores of the prevention of lymphedema in patients with postoperative breast cancer were analyzed by multiple linear regression analysis. Taking the total scores of knowledge, belief, and practice of lymphedema prevention in postoperative breast cancer patients as independent variables, along with the items showing statistical significance in the univariate analysis, self-expression, and hospital anxiety and depression scores as independent variables, multivariate step-by-step regression analyses were conducted with an entry-level *α* = 0.05 and the exclusion level α = 0.10. A *p* < 0.05 indicated that the difference was statistically significant.

## 3 Results

### 3.1 General information about the respondents

In this study, 321 questionnaires were distributed, of which 306 were valid questionnaires, with an effective recovery rate of 95.32%. The age of the survey respondents ranged from 28 to 78 years old, with a significant concentration in the age group of 40 to 59 years old (69.9%), the place of residence was mainly in the urban area (59.2%), the literacy level of 38.2% of the patients was junior high school, the highest percentage of average per capita monthly income of the family was in the range of 1,000–2,999 (30.1%), marriage status was mainly married (94.4%), the primary caregiver was the spouse (62.1%), the number of hospital admissions after surgery was 0–5 times (57.2%), 88.6% of the patients used medical insurance for medical expenses, 73.9% of the patients did not have lymphedema, 83.3% of the patients had been educated in the prevention of lymphedema, and the specific information is shown in Table 1.

**Table 1:** Results of univariate analysis of general information and prevention of lymphedema in postoperative breast cancer patients with knowledge, belief, and practice.

Event	Number of persons (%)	Total score of knowing, believing, and doing questionnaire (points, X ± S)	Knowledge dimension score (points, X ± S)	Belief dimension score (points, X ± S)	Practice dimension score (points, X ± S)
Age (years)					
20–39	43 (14.1)	54.65 ± 11.80	20.86 ± 7.13	16.44 ± 4.26	17.34 ± 5.26
40–59	214 (69.9)	54.16 ± 12.10	19.55 ± 7.48	17.03 ± 3.17	17.57 ± 4.85
60–78	49 (16.0)	51.67 ± 13.71	19.06 ± 7.95	16.44 ± 3.29	16.16 ± 6.21
F		0.72	0.99	1.50	0.92
*p*		0.48	0.37	0.22	0.39
Living condition					
Urban area	181 (59.2)	56.20 ± 11.69	21.25 ± 7.05	17.12 ± 3.60	17.81 ± 4.94
Townships	125 (40.8)	50.40 ± 12.47	17.33 ± 7.56	16.47 ± 2.95	16.60 ± 5.41
T		4.14	4.62	1.68	2.03
*p*		<0.001	<0.001	0.094	0.042
Educational attainment					
Primary and below	41 (13.4)	46.60 ± 14.00	15.97 ± 8.53	16.07 ± 3.37	14.56 ± 6.01
Junior high school	117 (38.2)	52.82 ± 11.09	18.52 ± 7.19	16.87 ± 3.39	17.42 ± 5.02
High school or junior College	74 (24.2)	56.40 ± 11.42	21.00 ± 6.63	17.29 ± 3.08	18.10 ± 4.73
College or bachelor’s degree	72 (23.5)	57.04 ± 12.53	22.33 ± 7.16	16.88 ± 3.57	17.81 ± 4.85
Graduate student or above	2 (0.7)	51.00 ± 8.48	15.50 ± 4.94	15.00 ± 2.82	20.50 ± 6.36
F		6.15	6.62	1.02	3.87
*p*		<0.001	<0.001	0.393	0.004
Careers					
Students	1 (0.3)	59.00	17.00	19.00	23.00
Workers	25 (8.2)	57.48 ± 10.73	20.28 ± 7.09	17.84 ± 2.54	19.36 ± 4.06
Farmers	55 (18.0)	50.76 ± 12.56	17.12 ± 7.52	16.69 ± 2.73	16.94 ± 5.81
Businessmen	8 (2.6)	52.50 ± 14.45	19.87 ± 9.37	15.62 ± 3.66	17.00 ± 4.98
Teachers	22 (7.2)	53.18 ± 8.8	18.86 ± 5.34	15.63 ± 3.10	18.68 ± 4.59
Medical personnel	14 (4.6)	56.50 ± 14.32	23.50 ± 7.83	17.35 ± 4.39	15.64 ± 5.61
Government staff	5 (1.6)	66.60 ± 13.70	27.60 ± 8.29	18.40 ± 4.33	20.60 ± 4.97
Services	20 (6.5)	55.75 ± 9.68	21.85 ± 6.20	16.20 ± 2.94	17.70 ± 3.58
Out of work	69 (22.5)	51.46 ± 11.90	18.04 ± 7.37	17.05 ± 3.14	16.36 ± 4.75
Else	87 (28.4)	55.24 ± 13.07	20.98 ± 7.52	16.90 ± 3.92	17.34 ± 5.55
F		1.83	2.76	1.00	1.45
*p*		0.062	0.004	0.437	0.163
Monthly per capita household income (Yuan)					
<1,000	53 (17.3)	48.98 ± 11.92	15.84 ± 7.57	16.98 ± 3.17	16.15 ± 4.83
1,000-2,999	92 (30.1)	53.21 ± 11.31	19.26 ± 6.66	17.02 ± 3.03	16.93 ± 5.19
3,000–4,999	96 (31.4)	54.01 ± 12.12	20.13 ± 7.19	16.75 ± 3.81	17.12 ± 5.34
5,000–9,999	50 (16.3)	60.16 ± 13.14	23.68 ± 7.68	17.12 ± 3.05	19.36 ± 4.77
≥10,000	15 (4.9)	52.60 ± 10.71	19.06 ± 7.24	15.26 ± 3.86	18.26 ± 4.69
F		5.78	7.82	1.01	2.99
*p*		<0.001	<0.001	0.401	0.019
Marital status					
Unmarried	1 (0.3)	86	40.00	18.00	28
Married	289 (94.4)	53.71 ± 11.97	19.55 ± 7.43	16.85 ± 3.28	17.29 ± 5.01
Divorced	11 (3.6)	57.18 ± 18.89	21.54 ± 8.60	17.45 ± 5.61	18.18 ± 7.57
Bereaved of spouse	5 (1.6)	47.20 ± 5.93	17.20 ± 2.77	15.40 ± 2.19	14.60 ± 6.42
F		3.092	2.929	0.464	2.014
*p*		0.027	0.034	0.707	0.112
Medical cost					
Own cost	35 (11.4)	52.88 ± 15.06	18.20 ± 9.06	17.14 ± 4.81	17.54 ± 5.88
Medical insurance	271 (88.6)	53.95 ± 11.96	19.84 ± 7.28	16.82 ± 3.13	17.29 ± 5.07
t		0.409	0.057	0.016	0.422
*p*		0.629	0.223	0.704	0.787
Caregiver					
Sons and daughters	60 (19.6)	51.20 ± 12.77	18.26 ± 8.10	16.68 ± 3.49	16.25 ± 5.46
Mate	190 (62.1)	54.35 ± 12.17	19.96 ± 7.38	16.81 ± 3.29	17.57 ± 4.87
Parents	23 (7.5)	54.60 ± 11.91	20.30 ± 7.46	16.26 ± 3.92	18.04 ± 4.75
Else	33 (10.8)	55.09 ± 12.57	19.93 ± 7.12	17.84 ± 3.06	17.30 ± 6.32
F		1.173	0.866	1.263	1.165
*p*		0.32	0.459	0.287	0.323
Number of hospital admissions after surgery					
0–5	175 (57.2)	52.88 ± 12.38	18.78 ± 7.48	16.73 ± 3.43	17.36 ± 5.32
6–10	76 (24.8)	56.52 ± 10.65	21.39 ± 6.90	17.19 ± 3.32	17.93 ± 4.62
11–20	28 (9.2)	53.42 ± 14.78	20.53 ± 8.18	16.75 ± 3.61	16.14 ± 5.14
21–	27 (8.8)	52.88 ± 13.25	19.48 ± 8.02	16.85 ± 2.81	16.55 ± 5.49
F		1.629	2.299	0.348	1.046
*p*		0.183	0.077	0.791	0.373
Whether lymphedema has occurred					
Yes	80 (26.1)	57.11 ± 12.12	22.51 ± 7.02	16.70 ± 3.49	17.90 ± 5.24
No	226 (73.9)	52.67 ± 12.22	18.64 ± 7.42	16.91 ± 3.32	17.11 ± 5.12
t		3.254	4.483	−0.571	1.591
*p*		0.006	<0.001	0.622	0.243
Whether or not they have received health education on prevention of lymphedema					
Yes	255 (83.3)	56.02 ± 11.07	21.20 ± 6.51	17.05 ± 3.13	17.76 ± 4.94
No	51 (16.7)	42.90 ± 12.57	11.92 ± 7.39	15.90 ± 4.23	15.07 ± 5.64
t		6.736	7.852	1.617	3.096
*p*		<0.001	<0.001	0.071	0.001

### 3.2 Knowledge, belief, and practice, self-representation, anxiety, and depression scores of postoperative breast cancer patients to prevent lymphedema

The results of this study showed that the lymphoedema prevention score for postoperative breast cancer patients was (53.83 ± 12.33), with knowledge (19.65 ± 7.58), belief (16.85 ± 3.36), and practice (17.32 ± 5.16), and total score rate of knowledge, belief, and practice was 58.51%, with the lowest knowledge score rate at 49.12%, the highest belief score rate at 70.20%, and the practice score rate was 61.85% (Table 2). The top 3 and bottom 3 scores of knowledge, belief, and practice are shown in Table 3. The self-expression scores of the postoperative breast cancer patients were (37.60 ± 7.04), which was in the middle level, the anxiety scores were (6.51 ± 3.20), and the depression scores were (5.37 ± 3.60), which were all asymptomatic.

**Table 2:** Knowledge, belief, and practice scores for prevention of lymphedema in postoperative breast cancer patients (*n* = 306).

Dimension	Minimum value	Maximum values	Score ( $\bar{X}$ ± SD)	Score rate (%)
Knowledge	0	40	19.65 ± 7. 58	49.12
Belief	0	24	16.85 ± 3. 36	70.20
Practice	0	28	17.32 ± 5. 16	61.85
Totals	15	91	53.83 ± 12.33	58.51

**Table 3:** Scores of knowledge, belief, and practice about prevention of lymphedema in 306 postoperative breast cancer patients for each dimension (top 3 and bottom 3 entries).

Entry	Element	Entry mean score ( $\bar{X}$ ± SD)	Score rate (%)
Top 3 of knowledge dimensions	Do you know where you should go for examination and treatment after developing lymphedema?	2.17 ± 0.90	54.25
	Do you know the medical maneuvers to avoid in the affected arm after surgery?	2.16 ± 0.86	54.00
	Do you know how to avoid skin damage to the affected upper extremity?	2.03 ± 0.86	50.75
Bottom 3 of knowledge dimension	Do you recognize early lymphedema?	1.82 ± 0.91	45.50
	Did you know Daily Precautions for Preventing Upper Extremity Lymphedema?	1.85 ± 0.94	46.25
	Will you assess your skin and swelling on the affected arm?	1.89 ± 0.90	47.25
Top 3 of belief dimensions	You need to pay attention to the protection of your limbs and a healthy lifestyle.	2.92 ± 0.61	73.00
	Do you think prevention is better than cure for upper extremity lymphedema?	2.82 ± 0.71	70.50
	You’re confident you are doing a good job of preventing upper extremity lymphedema	2.81 ± 0.67	70.25
Bottom 3 of belief dimension	Knowledge of prevention methods can prevent upper extremity lymphedema	2.69 ± 0.78	67.25
	Adherence to standardized functional exercises prevents upper extremity lymphedema	2.78 ± 0.69	69.50
	Learning to recognize and assess lymphedema conditions early can help with lymphedema prevention and treatment	2.81 ± 0.63	70.25
Top 3 of practical dimensions	You can control your weight with a balanced diet	2.62 ± 0.93	65.50
	You emphasize a healthy lifestyle and protection of the affected arm.	2.61 ± 0.93	65.25
	When you have questions about lymphedema, you’ll reach out to your healthcare provider promptly.	2.54 ± 0.99	63.5
Bottom 3 of practical dimensions	You continue to perform functional exercises daily	2.39 ± 0.97	59.75
	You will be motivated to care and learn about upper extremity lymphedema	2.32 ± 0.99	58.00
	You check the skin on the affected side daily for damage and swelling of the arm	2.41 ± 1.04	60.25

### 3.3 Univariate analysis of knowledge, belief, and practice scores for prevention of lymphedema in postoperative breast cancer patients

The results of the study showed that the differences in the knowledge scores on lymphedema prevention among postoperative breast cancer patients with different places of residence, literacy, occupation, per capita monthly family income, marital status, whether lymphedema had occurred, and whether or not they had received the knowledge education on lymphedema prevention were statistically significant (*p* < 0.05). Comparison of belief scores on prevention of lymphedema among postoperative breast cancer patients with other demographic characteristic factors showed no statistically significant difference (*p* > 0.05). Comparison of practice scores for lymphedema prevention among postoperative breast cancer patients with various places of residence, literacy level, per capita monthly household income, and whether or not they had received health promotion for lymphedema prevention, the difference was statistically significant (*p* < 0.05). Data are shown in Table 1.

### 3.4 Analysis of the correlation between knowledge, belief, and practice of preventing lymphedema with self-representation, anxiety, and depression in postoperative breast cancer patients

The results of Spearman correlation analysis showed that there was a two-by-two correlation between the knowledge, belief, and practice of lymphedema prevention and the total scores of knowledge and belief of postoperative breast cancer patients, and there was a significant positive correlation between knowledge and belief of lymphedema prevention and self-exposure level scores (*p* < 0.01). There was a negative correlation between knowledge and belief of lymphedema prevention and the scores of anxiety and depression (*p* < 0.01), as shown in Table 4.

**Table 4:** Correlational analysis of the dimensions of knowledge, belief, and practice in the prevention of lymphedema in postoperative breast cancer patients with self-representation, anxiety, and depression (*n* = 306).

Variable pair	ρ	95% CI	*p*-value
Knowledge vs. belief	00.294	[0.185, 0.397]	<0.001**
Belief vs. practice	00.222	[0.109, 0.329]	<0.002**
Knowledge vs. practice	00.454	[0.355, 0.543]	<0.001**
Belief vs. depression	−0.171	[−0.279, −0.057]	<0.014*
Knowledge vs. depression	−0.241	[−0.346, −0.129]	<0.001**
Practice vs. depression	−0.387	[−0.481, −0.285]	<0.001**
The total score vs. knowledge	00.879	[0.846, 0.901]	<0.001**
The total score vs. belief	00.545	[0.458, 0.620]	<0.001**
Total score vs. practice	00.756	[0.701, 0.800]	<0.001**
The total score vs. self-expression	00.261	[0.150, 0.363]	<0.001**
The total score vs. anxiety	−0.311	[−0.409, −0.202]	<0.001**
Total score vs. depression	−0.355	[−0.449, −0.249]	<0.001**
Self-expression vs. knowledge	00.268	[0.157, 0.371]	<0.001**
Self-expression vs. practice	00.283	[0.172, 0.385]	<0.001**
Self-expression vs. depression	00.290	[0.179, 0.391]	<0.001**
Self-expression vs. anxiety	00.106	[−0.007, 0.216]	0.067

**p < 0.01; *p < 0.05.

### 3.5 Multiple linear regression analysis of the factors influencing the knowledge and belief about the prevention of lymphedema in postoperative breast cancer patients

The assigned values of the independent variables are shown in Table 5.

**Table 5:** Method of assigning values to the independent variables of the factors.

Independent variable	Description of the assignment
Living condition	City = 1, Township = 0
Educational attainment	1 = Elementary school, 2 = Middle school, 3 = High school, 4 = College or bachelor’s degree, 5 = Graduate school or higher
Marital status	Married = 0, unmarried/divorced/widowed = 1
Monthly per capita household income	1 = <1,000, 2 = 1,000–2,999, 3 = 3,000–4,999, 4 = 5,000–9,999, 5 = ≥10,000
Whether or not they have received health education on the prevention of lymphedema	Yes = 1, No = 0
Whether lymphedema has occurred self-expression Anxiety Depression	Yes = 1, No = 0 Continuous variable Continuous variable Continuous variable

This study systematically explored the influencing factors and mechanisms of knowledge, belief, and practice (KBP) regarding lymphedema prevention among postoperative breast cancer patients through multiple linear regression analysis. The findings revealed that KBP levels are influenced by multidimensional factors, with preventive education being the core driving factor: patients who received preventive education showed a significant increase in knowledge scores by 7.828 points (accounting for 35.5% of the total variance), an improvement in behavior scores by 1.983 points, and a rise in total scores by 11.330 points, indicating that preventive education effectively enhances patients’ cognitive levels and promotes the formation of healthy behaviors. Anxiety, in contrast, exhibited a significant negative correlation with KBP levels: for every 1-point increase in anxiety, knowledge, behavior, and total scores decreased by 0.358, 0.472, and 0.636 points, respectively. This suggests that anxiety may hinder patients’ self-management abilities by depleting cognitive resources, reducing motivation, and triggering behavioral avoidance. Depression demonstrated a paradoxical effect: while it facilitated knowledge acquisition (+1.887 points), it led to a decrease in total scores by 0.565 points, reflecting its characteristic of “high knowledge but low practice”—despite patients’ grasp of relevant knowledge, emotional burden inhibited the translation of knowledge into behavior. Additionally, self-disclosure and household income played important supportive roles by enhancing confidence (knowledge +0.170 points/behavior +0.110 points) and improving resource accessibility (total score +1.562 points). The results show that whether to receive knowledge of lymphedema prevention health education, per capita monthly family income, and anxiety and depression levels are critical influencing factors on the level of knowledge, belief, and practice of patients after breast cancer surgery (*p* < 0.05), as shown in Table 6.

**Table 6:** Multiple linear regression analysis of factors influencing the scores of knowledge, belief, and practice.

Implicit variable	Independent variable	Regression coefficient	Standard error	Standardized regression coefficient	*T*-value	*p*-value
Knowledge	(Constant)	4.853	2.240	–	2.166	0.031
	Received health education on the prevention of lymphedema	7.828	0.954	0.389	8.206	<0.001
	Monthly per capita household income	0.948	0.341	0.139	2.783	0.006
	Apprehensive	−0.358	0.111	−0.153	−3.225	0.01
	Self-expression	0.170	0.051	0.160	3.334	0.01
	Lymphedema has occurred.	2.338	0.804	0.137	2.906	0.004
	City district	1.887	0.756	0.124	2.497	0.013
Practice	(Constant)	14.080	1.742	-	8.082	<0.001
	Anxiety	−0.472	0.077	−0.329	−6.087	<0.001
	Self-expression	0.110	0.040	0.150	2.757	0.006
	Received health education on prevention of lymphedema	1.983	0.721	0.143	2.749	0.006
The total score	(Constant)	39.267	3.987	–	9.848	<0.001
	Received health education on prevention of lymphedema	11.330	1.605	0.343	7.057	<0.001
	Despondent	−0.565	0.223	−0.165	−2.530	0.012
	Monthly per capita household income	1.562	0.553	0.139	2.826	0.005
	Anxiety	−0.636	0.241	−0.165	−2.634	0.009
	Self-expression	0.219	0.090	0.125	2.426	0.016

Knowledge is the dependent variable R^2^ = 0.355, adjusted R^2^ = 0.342, F = 27.428, *p* < 0.001; practice is the dependent variable R^2^ = 0.194, adjusted R^2^ = 0.186, F = 24.301, *p* < 0.001; Knowledge- Belief -Practice is the dependent variable R^2^ = 0.308, adjusted R^2^ = 0.296, F = 26.649, *p* < 0.001.

## 4 Discussion

### 4.1 The levels of knowledge, belief, and practice of lymphedema prevention need to be improved

#### 4.1.1 Current knowledge on prevention of lymphedema in postoperative breast cancer patients

The knowledge score of postoperative breast cancer patients preventing lymphedema was (19.65 ± 7.58), and the scoring rate was 49.12%, which was at a low level according to the classification criteria, indicating that the knowledge level of preventing lymphedema in postoperative breast cancer patients needs to be improved. In this study, 83.3% (255/306) of the patients received knowledge of lymphedema prevention. However, the level of knowledge, belief, and practice of lymphedema prevention was still at the low-to-middle level, which may be because 40.8% of the subjects included in this study were from urban areas, and 51.6% of the patients’ literacy level was less than junior high school. Hence, the patient’s education level and the acceptance of health education were low. They were insufficiently cognizant of lymphedema prevention and had a low ability to accept information, and tended to accept information after suffering from illness. They do not communicate effectively with healthcare personnel, so they cannot understand the knowledge related to the disease, and they are more likely to forget the newly accepted health education knowledge (12). A study by Borman et al. similarly found that only a small proportion of patients (19%) in developing countries received information and education about lymphedema and that there was an unmet need for education or information about lymphedema after breast cancer treatment. The majority of breast cancer patients who participated in the study reported that they were unaware of lymphedema before it developed, did not notice the onset of symptoms, and did not take any preventive measures (13) The results of the further analysis found that the three lowest-scoring items were “early identification of lymphedema in postoperative breast cancer patients,” “assessment of the skin and swelling of the affected arm,” and “daily precautions for the prevention of upper limb lymphedema.” The above results may also be related to the following reasons: (1) 58.6% of the patients in this study were admitted to the hospital 0–5 times after surgery, and this group of patients may be more concerned about the treatment of breast cancer itself rather than the prevention of lymphedema (14); (2) At present, many medical institutions have not standardized the management of lymphedema prevention, and primary prevention measures such as measurement of the circumference of both upper limbs and high-risk screening for lymphedema are not strictly implemented in perioperative breast cancer patients, resulting in the majority of patients not being able to assess the skin of the affected arm and its swelling; (3) healthcare personnel do not have sufficient knowledge of lymphedema, and the level of health promotion knowledge is insufficient (15–17). The early identification of lymphedema is relatively more specialized, and the teaching method is rather simple, with insufficient depth and breadth, so the patients are not fully educated; (4) although health knowledge teaching is carried out, without timely tracking and continuous feedback on the effect of health education, the impact of health education is not satisfactory. It has been reported that some patients who received information about lymphedema were not satisfied with health education (17). The above data suggest that medical institution administrators need to strengthen the standardized management of lymphedema further, improve the knowledge reserve of medical personnel, strengthen the risk education of lymphedema at the initial stage of breast cancer treatment, improve risk awareness, timely understand the obstacles faced by patients and their needs, and pay attention to the health education methods of breast cancer-related lymphedema (18). According to the content of health education, we should choose the way that is easy to be accepted by patients for health education, use the teach-back method or narrative method to timely understand the level of knowledge, belief, and practice of postoperative breast cancer patients in the prevention of lymphedema, and correct the corresponding practice in time, to effectively improve the level of knowledge of the patients in the prevention of lymphedema (19). The knowledge level of the patients in preventing lymphedema can be effectively enhanced (20).

#### 4.1.2 Status of belief toward prevention of lymphedema in postoperative breast cancer patients

The belief of postoperative breast cancer patients toward preventing lymphedema scored (16.85 ± 3.36), with a score rate of 70.20%, which is at a medium level according to the classification criteria, indicating that the belief still needs to be improved. The analysis of the six entries of the belief dimension shows that “attention to the protection of the affected limb and a healthy lifestyle” has the highest score, indicating that postoperative breast cancer patients pay much attention to protecting the affected limb and a healthy lifestyle. In contrast, “knowledge of prevention methods can prevent the occurrence of upper limb lymphedema” scored the lowest, which is consistent with the score of the knowledge dimension. The reason might be due to the lack of knowledge and the lack of awareness of the risk of lymphedema, which did not attract great attention from the patients, and then affected the patients’ belief (21).

#### 4.1.3 Current practice status of prevention of lymphedema in postoperative breast cancer patients

The analysis of the seven entries in the practice dimension of the survey showed that the entry with the highest score in the practice dimension was “focusing on a healthy lifestyle and protection of the affected arm,” which was consistent with the scores in the knowledge and belief dimensions. The three lowest scores were “daily adherence to functional exercises,” “active attention to and learning about upper limb lymphedema,” and “daily inspection of the affected side for skin damage and arm swelling.” The reason is that patients’ adherence to functional exercises gradually decreases as the function of the affected limbs recovers after surgery, and the medical staff focuses their attention on patients at the initial stage of postoperative treatment of breast cancer, and their attention to the affected limbs gradually decreases in the later stages of the treatment process, and they seldom follow up the patients in the long term (22). The lack of adequate supervision and benign support made it difficult for patients to adhere to daily functional exercises and check the skin and arm condition of the affected arm. With the shortening of the average hospitalization day, patients spend most of their time at home for self-care and lack a communication platform with medical staff to obtain relevant information after discharge from the hospital. Therefore, a hospital-home linkage BCRL multidisciplinary management model for postoperative breast cancer can be implemented in clinical work to standardize patients’ practice (23).

### 4.2 Analysis of the correlation between knowledge and belief about the prevention of lymphedema in postoperative breast cancer patients

According to the theory of knowledge, belief, and practice, the change of human practice is divided into three processes: having the proper knowledge and thinking positively, gradually forming belief, and then acting positively. Knowledge is the basis of practice change, and belief/attitude is the driving force of practice change (24). After correlation analysis, it is concluded that there is a positive correlation between the knowledge, belief, and practice of postoperative breast cancer patients in preventing lymphedema. This result indicates that the higher the knowledge of postoperative breast cancer patients about lymphedema prevention, the more positive the belief toward lymphedema prevention and the better practice, consistent with the conceptual model of knowledge, belief, and practice. Therefore, it is important to strengthen the training of patients’ understanding of lymphedema prevention, continuously evaluate patients’ knowledge, belief, and practice, timely correct patients’ misperceptions and bad habits, enhance patients’ correct health belief, and improve breast cancer patients’ adherence to lymphedema prevention.

### 4.3 The current status of knowledge, belief, and practice is influenced by a variety of factors

#### 4.3.1 Postoperative breast cancer patients who have been educated about lymphedema prevention have higher levels of knowledge, belief, and practice about lymphedema prevention

The findings of this study demonstrated that exposure to lymphedema prevention health education significantly influenced knowledge and practice scores. The correlations among Knowledge, Belief, and Practice were statistically significant, albeit generally weak to moderate in strength. Notably, the strongest association was observed between Knowledge and Practice (*ρ* = 0.454, 95% CI [0.355, 0.543]), indicating knowledge’s substantial impact on behavioral outcomes. This suggests that enhancing patients’ understanding of lymphedema prevention may be a critical driver for improving adherence to self-care practice.

Our findings demonstrate that while health education significantly improved patients’ knowledge scores (+7.828 points), the absolute level remained suboptimal (mean 19.65/40, SD = 3.962), with a mere 19% knowledge-to-behavior conversion rate, indicating limitations in current educational paradigms. At present, the health education level of patients in China has not been comprehensively improved, and optimizing knowledge education on lymphedema prevention has become one of the key work directions of the China Anti-Cancer Association, and it is also the core goal of the subsequent practice of this study.

To systematically enhance patient education efficacy, we propose a multidimensional strategy: First, comprehensive health education and disease popularization intervention are implemented in the admission stage for patients. Through diversified media (such as brochures, popular science videos, face-to-face explanations, etc.), we ensure that patients understand preventive measures. The education content should be simple and practical, such as designing concise prevention guidelines highlighting key daily measures, such as avoiding limb injuries and keeping the skin clean. Second, to build a multilevel and multiplatform education model. Based on the patient’s knowledge level and individualized needs, the stepped education content is designed to gradually transition from basic knowledge popularization to behavior guidance and psychological support. Integrate online and offline resources (such as hospital public accounts, short video platforms, online courses, etc.) to expand the coverage of education and enhance its pertinence. For example, for young patients, vivid content can be provided through short video platforms, and face-to-face presentation or paper materials for older adults patients. In addition, mobile applications can be developed to provide personalized content and reminders. Third, the content and form of education should be optimized to improve patients’ participation and practical ability. Scientific and practical content design (such as illustrated materials, peer support groups, etc.) enhances the attraction of educational content. Interactive education methods (such as scenario simulation, role playing, etc.) are introduced to help patients master prevention skills in simulation practice. For example, practice wearing pressure clothes or body massage correctly through situational simulation. At the same time, group discussions or workshops are organized to consolidate the learning effect through peer support and experience sharing. Now the education method has failed to meet the standard, BCRL education should be based on the needs of survivors and the preferred learning way for personalized learning. This gets positive feedback from the patient (25, 26).

#### 4.3.2 Postoperative breast cancer patients with high monthly household income have higher levels of knowledge, belief, and practice about lymphedema prevention

Patients with higher monthly household incomes have better family and social conditions, enjoy more medical resources, have wider access to relevant knowledge, and can easily acquire knowledge related to lymphedema prevention. This suggests that healthcare professionals should pay more attention to patients with low monthly household income, assess their needs for disease-related knowledge, and guide them to make full use of information technology, such as the internet and electronic devices, to strengthen their ability to acquire information resources (27). In addition, healthcare professionals should strengthen health promotion efforts to improve patients’ knowledge of lymphedema prevention, correct misperceptions, establish positive belief, and promote healthy practice.

#### 4.3.3 A good level of self-expression is conducive to improving the level of knowledge, belief, and practice in preventing lymphedema in postoperative breast cancer patients

This study found that the level of self-representation of postoperative breast cancer patients is at a moderate level, and the stronger the level of self-representation of postoperative breast cancer patients, the higher the level of lymphedema prevention knowledge belief, and practice. Cognitive processing theory suggests that self-expression is the process of transforming ambiguous ideas and events in the mind into concrete speech and that continuous self-expression can enable individuals to process the information re-cognitively, thus allowing them to redefine adverse events and change the way of thinking about them, reduce negative emotions, and realize the reconstruction of cognitive structure. Self-expression can also lead to more social support, more timely help, and ultimately improved health status (28). A meta-analysis conducted by Yao et al. found that self-disclosure can enhance patients’ confidence in coping with their illness, facilitate better adaptation to social roles, and help them gain social support, thereby contributing to improved disease management in the future (29). Patients with strong self-disclosure ability are more likely to communicate effectively with medical staff to acquire correct knowledge and guidance, to improve self-care compliance. Conversely, patients with weak self-disclosure may be unable to effectively implement preventive measures due to insufficient information access or misunderstanding (30). Therefore, throughout the diagnosis treatment, and rehabilitation stages of breast cancer patients, we can provide patients with opportunities for self-expression through various interventions, such as written expression, verbal expression, group expression, and husband-to-wife expression, to improve the level of postoperative breast cancer patients’ expression and enhance the level of knowledge, belief, and practice of lymphedema prevention.

#### 4.3.4 Anxiety and depression levels as influential factors on knowledge, belief, and practice

Correlation analysis showed that anxiety and depression scores were significantly negatively correlated with the total score of knowledge, belief, and practice, and the higher the anxiety and depression scores, the worse the level of lymphedema prevention practice. Anxiety and depression are persistent negative state of mind, which manifests themselves in different degrees of sadness, sorrow, tension, fear, and uneasiness, and are capable of eliciting physiological, emotional, intellectual, social, and spiritual responses in individuals. Anxiety and depression may influence patient self-care behavior through multiple mechanisms. Anxiety may lead to excessive concerns about lymphedema, leaning the patients to rely on medical interventions and ignore the importance of self-care. For example, anxious patients may be much concerned about negative information because of the development of lymphedema and ignoring positive precautions. Depression may reduce patients’ self-efficacy and make them less motivated to implement daily precautions. By psychological intervention, the adherence to self-management among lymphedema patients can be improved. This finding is consistent with the research results of Shen et al., as demonstrated in a theory-based qualitative study (31). The higher the scores of anxiety and depression, the worse the patient’s psychological state is, and the lower the self-efficacy level is, which affects the patient’s level of knowledge, belief, and practice in preventing lymphedema. It is recommended that healthcare professionals pay attention to the psychological state of patients and incorporate emotional factor assessments into breast cancer health education and support programs. Through cognitive-behavioral interventions, mindfulness-based stress reduction therapy, or psychological education, patients can be helped to face the disease positively, thereby enhancing their self-efficacy and psychological adaptation (27, 32).

## 5 Limitations

The limitations in this study include the use of a non-probability design method. In contemporary clinical research, hospitals increasingly enforce stringent data protection protocols to comply with evolving ethical guidelines and institutional review board requirements. These privacy-preserving measures—while ethically imperative—substantially restricted access to comprehensive patient registries across multiple centers. Despite efforts to maximize data diversity through extended recruitment and multisource collection, it restricted access to comprehensive patient demographics. Consequently, data collection was limited to several Grade A tertiary hospitals (China’s highest-tier medical institutions) where the research team maintained collaborative agreements, inherently narrowing population diversity.

## 6 Conclusion

This study used a cross-sectional investigation and multidimensional data analysis to investigate the interaction of knowledge, belief, behavior, and psychosocial factors in lymphedema prevention in patients after breast cancer surgery. It is found that there are significant obstacles in the transformation of knowledge into behavior, which need to be solved by a multidimensional intervention. Specifically, the level of knowledge and action was moderately negatively correlated with depression and anxiety, suggesting the importance of mental health in the transformation of behavior; the improvement of self-disclosure level can significantly promote the improvement of the overall knowledge, belief and action level, indicating that the comorbidities of physical and mental symptoms need collaborative management. In addition, low-income groups have a lower level of knowledge and practice due to economic pressure and insufficient medical resources. Based on this, it is suggested that medical institutions should strengthen the standardized management of lymphedema, pay attention to low-income groups, strengthen the training of medical staff, and promote behavioral transformation through structured education. At the same time, psychological assessment should be included in health education programs, combined with continuous hospital-home follow-up services, to improve patients’ cognitive level and health beliefs.

## Data Availability

The raw data supporting the conclusions of this article will be made available by the authors, without undue reservation.
